# Legal Uncertainty—The Gray Area around Substandard Medicines: Where Public Health Meets Law

**DOI:** 10.4269/ajtmh.19-0645

**Published:** 2019-11-18

**Authors:** Eugenia Olliaro, Piero Olliaro, Calvin W. L. Ho, Raffaella Ravinetto

**Affiliations:** 1 Independent Researcher, Geneva, Switzerland;; 2 Centre for Tropical Medicine and Global Health, Nuffield Department of Medicine, University of Oxford, Oxford, United Kingdom;; 3 Centre for Biomedical Ethics, Yong Loo Lin School of Medicine, National University of Singapore, Singapore;; 4 Institute of Tropical Medicine, Antwerp, Belgium

## Abstract

A vicious circle links lack of equitable access to health to the supply of poor-quality medicines, which amount to one-tenth of medicines available in low- and middle-income countries. The WHO introduced a new, public health–focused definition of substandard and falsified (SF) medicines, which offers opportunities for governments to broaden the scope of interventions to combat poor-quality medicines. At the same time, translating it into legal and regulatory measures may be challenging because this definition is not free of ambiguity (in that, there is a gray area between intentionally falsified and unintentional substandard medicines), and some countries may not have appropriate regulatory mechanisms/jurisdictions in place. The focus of the article is to consider what a public health–informed legal and regulatory environment could look like in light of WHO’s SF definition and propose appropriate measures to put it into effect. We present a “legal levers matrix” that may assist legislators and policymakers evaluate the adequacy of measures (i.e., criminal, civil, and administrative mechanisms) to address the problem of poor-quality medicines, particularly in terms of their configuration. In addition, this matrix underscores the importance of fostering dialogue between medical/public health and the legal/regulatory communities and to develop alternative/complementary solutions, including regulatory strengthening and nonpunitive actions. Substandard and falsified medicines arise from the interplay between societies, economies, and behaviors: effective regulation is necessary to disincentivize the production and/or supply of SF medicines, whereas health systems should strive to provide affordable medicines to all levels of society.

## INTRODUCTION

The WHO estimates that on average, 10.5% of the medicines in low- and middle-income countries (LMICs) are of poor quality,^1^ failing to meet standards for amounts of active ingredients(s), impurities, bioavailability, sterility, stability, packaging, etc.^2,3^ They threaten the welfare of individuals and societies, undermine health systems, and challenge the achievement of universal health coverage (UHC), which requires equitable access to quality-assured medicines.^4–6^ Collectively, poor-quality medicines are now called “substandard and falsified (SF)” medical products (Table 1) as per a 2017 World Health Assembly (WHA) resolution.^7^ “Falsified” implies *intent of deceiving*, whereas “substandard” medicines are *authorized, but noncompliant with quality standards*. This is a welcomed improvement over the previous complex and disputed “working definition” of “substandard/spurious/falsely-labelled/falsified/counterfeit medical products,” as it squarely focuses on public health. It now needs to be translated into action in countries to prevent, correct, compensate for, and, where necessary, sanction wrongdoings.

**Table 1:** WHO and working definitions

	WHO definitions	Working definitions
Substandard	Substandard medical products^7^	
	Also called “out of specification,” these are authorized medical products that fail to meet either their quality standards or their specifications, or both.	
	Note: When the authorized manufacturer deliberately fails to meet these quality standards or specifications due to misrepresentation of identity, composition, or source, then the medical product should be considered “falsified.”	
Falsified	Falsified medical products^7^	
	Medical products that deliberately/fraudulently misrepresent their identity, composition or source.	
	Any consideration related to intellectual property rights does not fall within this definition.	
	Such deliberate/fraudulent misrepresentation refers to any substitution, adulteration, reproduction of an authorized medical product or the manufacture of a medical product that is not an authorized product.	
	“Identity” shall refer to the name, labelling or packaging or to documents that support the authenticity of an authorized medical product.	
	“Composition” shall refer to any ingredient or component of the medical product in accordance with applicable specifications authorized/recognized by NRRA.	
	“Source” shall refer to the identification, including name and address, of the marketing authorization holder, manufacturer, importer, exporter, distributor or retailer, as applicable.	
	Medical products should not be considered as falsified solely on the grounds that they are unauthorized for marketing in any given country.	
Unregistered	Unregistered/unlicensed medical products^7^	
	Medical products that have not undergone evaluation and/or approval by the NRRA for the market in which they are marketed/distributed or used, subject to permitted conditions under national or regional regulation and legislation.	
Negligence		A failure to behave with the level of care that someone of ordinary prudence would have exercised under the same circumstances. The behavior usually consists of actions, but can also consist of omissions when there is some duty to act (e.g., a duty to help victims of one's previous conduct, or a duty to take the precautions that are knowingly needed when performing a given operation).^21^
Legal authorities		Any regulatory, administrative, legislative, executive and judicial authority that adopt, apply and enforce, respectively, national and international regulations and laws (authors’ ad hoc definition).
National Medicines Regulatory Authority (NMRA)	Effective medicines regulation promotes and protects public health by ensuring that:	Note: NMRA are created and regulated by administrative law (see Table 2).
	medicines are of the required quality, safety and efficacy;	
	medicines are appropriately manufactured, stored, distributed and dispensed;	
	illegal manufacturing and trade are detected and adequately sanctioned;	
	health professionals and patients have the necessary information to enable them to use medicines rationally;	
	promotion and adverting is fair, balanced and aimed at rational drug use;	
	access to medicines is not hindered by unjustified regulatory work.	
	National governments are responsible for establishing strong NMRAs with clear mission, solid legal basis, realistic objectives, appropriate organizational structure, adequate number of qualified staff, sustainable financing, access to up-to-date evidence based technical literature, equipment and information, capacity to exert effective market control. Medicines regulatory authorities must be accountable to both the government and the public and their decision-making processes should be transparent. Monitoring and evaluation mechanisms should be built into the regulatory system to assess attainment of established objectives.^22^	
Poor-quality medical products/medicines		A term inclusive of falsified, substandard, and degraded medicines, as well as of any medicines that fail chemical analysis but for which there is insufficient information to assign them to one of these subgroups^7^
Natural and legal persons		Persons having legal status as individuals, and corporate bodies, companies, or other entities respectively, which have legal rights and are subject to obligations.^23^
Intent		The mental desire to act in a particular way, or the state of mind that determines an explicit choice to adopt a behaviour that could lead to regulatory, civil or legal sanctions.^24^
Willingness		Synonym of intent
Legal levers matrix		A proposed guidance to translate the WHO definition of SF medicines into national laws and provisions. It includes a public health-informed legal levers matrix to define to which extent civil and criminal provisions apply at national level and which type of sanctions relate (authors’ ad hoc definition).

SF = substandard and falsified. The definitions are either taken verbatim from relevant WHO documents and reports or adopted ad hoc for this work.

**Figure 1.**
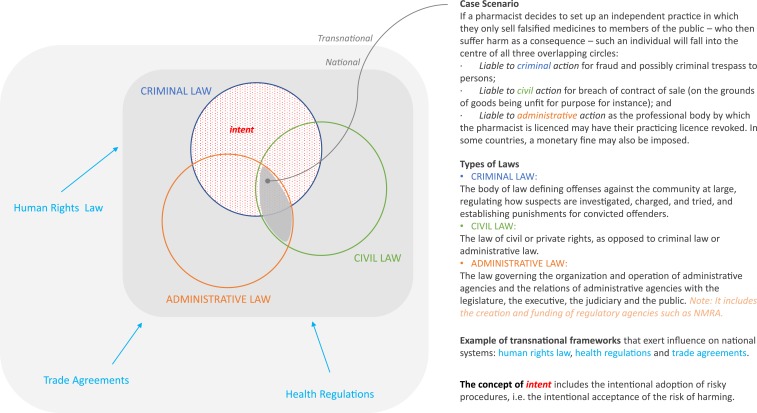
Legal levers matrix.

This article is about how national legal and regulatory frameworks may be arranged or configured in terms of their administrative, civil, and criminal levers to better support the goals that underlie the revised SF definition of WHO (Table 2). We explore ambiguities between deliberate and unintentional poor-quality medicines and reflect on a public health–informed “legal levers matrix” (Figure 1) to help legislators and policymakers navigate the intersection of regulatory enforcement and civil/criminal prosecution. This article is not about WHO’s third category (“unregistered/unlicensed medical products”) as it is unambiguous because introducing a medicine in a country without regulatory clearance is always a regulatory breach. It is also not about infringements of intellectual property law, particularly patents or copyrights.

**Table 2:** Types of laws and actions applicable to substandard and falsified (SF) medicines

Type of Law	Definition	Errors/Wrongs	Actions
Administrative law	The law governing the organization and operation of administrative agencies (including executive and independent agencies) and the relations of administrative agencies with the legislature, the executive, the judiciary and the public.^25^	Administrative error	(Minor error) A fine may be imposed or products may be ceased, typically as a reprimand to ensure that procedures are put in place to ensure that the error is not repeated. Other corrective orders may be imposed by a regulator.
	Note: Public health law is a branch of administrative law. Public health practice is governed by the rules, procedures and principles of administrative law. As legal background, administrative law itself is a branch of public law.^26^		(Serious error) A regulatory authority has the power to stop production and force the manufacturing plant or business to be closed.
Civil law	The law of civil or private rights, as opposed to criminal law or administrative law.^25^	Civil wrong	Civil action may be brought for compensation, that tends to be restitutive.
Criminal law	The body of law defining offenses against the community at large, regulating how suspects are investigated, charged, and tried, and establishing punishments for convicted offenders.^25^	Criminal wrong	Criminal charges may be pressed, and the penalty could be fine and/or imprisonment imposed on certain individuals.
International Law	The body of legal rules, norms, and standards that apply between sovereign states and other entities that are legally recognized as international actors.	International wrong	International sanctions may apply, including trade sanctions.
			*It is important to stress that SF medicines must not be confused with intellectual property protection and related infringements of trade law.*

The definitions either come from law dictionaries or have been adopted ad hoc for this work.

## REGULATORY ENFORCEMENT AND CIVIL/CRIMINAL PROSECUTION: COMPLEMENTARY ROLES

Medicine quality is governed through National Medicines Regulatory Authorities (which are regulated by administrative law, see Table 1)—ensuring medicines approved for use are effective, safe, and quality-assured^8^—and national civil/criminal prosecution authorities—enforcing civil and criminal laws to regulate behaviors and compensate/sanction wrongdoings. These authorities need to be supported by an adequate legal framework. Inevitably, laws about SF medicines vary broadly across countries (E. Olliaro, unpublished data), and do not always cover the range of cases arising.

A key challenge in applying the new WHO definition is that the definition of “substandard” could lead to systematically equate a poor-quality medicine produced by the owner of a marketing authorization with a nondeliberate mistake. However, there are gradations for “substandard”: substandard batches might result from accidental errors, negligence, or from *systematically and deliberately* implementing poor manufacturing standards. Although equally damaging from a public health standpoint, legally this distinction is critical; however, demonstrating deliberate intent to implement poor standards and to mislead regulatory supervision is often challenging.

Three illustrative examples are as follows. 1) Miltefos^®^, a local generic mandated by the Bangladesh government because the brand name miltefosine was unavailable and unaffordable,^9^ was found to contain no miltefosine, resulting in patients dying of untreated visceral leishmaniasis.^10^ Despite an existing body of law,^11^ basic quality checks did not take place, and it remains unclear whether Miltefos^®^ (Bangladesh) was a falsified or a substandard product resulting from a production error or negligence. 2) Isotab^®^ (**Efroze Chemical Industries, Karachi, Pakistan)**, a product containing isosorbide mononitrate produced in Pakistan, caused deaths and serious toxicities following contamination with pyrimethamine.^12^ Although this might have been an accident, a legal investigation concluded that “the first and the foremost responsibility for lapses lies on [the company], its top management and its officials, who had a legal obligation to ensure that …. the drug was manufactured and tested in strict compliance with the current Good Manufacturing Practices”.^13^ 3) A Chinese vaccine maker, Changsheng Biotechnology, was found to have fabricated production and inspection records and to have arbitrarily changed process parameters and equipment during production of human rabies vaccines, which was treated as “falsification.” Conversely, intent to deceive was not proven for “substandard” diphtheria, pertussis, and tetanus vaccines by the same company.^14^ Under the revised SF definition, the requisite intent has been broadened to better enable regulatory and compensatory actions to be taken by the state.

## FROM WHO DEFINITIONS TO NATIONAL LEGISLATIONS

Guidance is needed on how to consistently interpret SF definitions, to orient appropriate and adequate legal actions and, when required, sanctions. We propose a “legal levers matrix” that integrates the concepts of “intent” along with “consequences,” public interest, fairness, and justice to provide guidance to policymakers on legal levers that need to be in place, and the extent that they should be implemented.

### Determinants of legal intervention.

Whereas in other fields a “liability scale” is applied to natural and legal persons who caused harm either accidentally, by negligent behaviors or by a primary intent to harm, in pharmaceutical production, the concept of *intent* is not unequivocally defined. Poor manufacturing practices—a frequent cause of poor-quality medicines—can be “unintentional” or be adopted “intentionally.” Some manufacturers modulate their quality assurance system to the regulatory capacity of the country of destination ^2^ to lower production costs, reducing standards “to an extent that still complies with local regulatory requirements […]”^15^ or with the capacity of local regulators to detect insufficient standards. *Harming* is not the primary intent of such practices, but the primary intent (cutting costs) implies *accepting the risk of harming*.

### Strengthening legal and regulatory capacity to intervene appropriately.

Views diverge as to how the SF problem should be tackled legally. Attaran proposed a “Model Law on Medicine Crime” as a universal legal framework for medicine crimes^16^ proceeding with liability steps and proposing aggravation and mitigation principles. The *consequences* of the offense would define the sanctions, but manufacturers who were knowingly adopting substandard practices would either way be held accountable. Whereas Attaran’s model focuses primarily on criminal law, ‘t Hoen and Pascual call for focusing on the protection of individual and public health by promoting high-quality standards in generic industry, technical assistance to (local) manufacturers, and secure supply chains.^17^

Both views have their own merits and limitations and should be viewed as complementary. Stringent regulation, unfortunately as yet unattained for 74% of WHO member states,^18^ is essential to prevent poor-quality medicines. It should be complemented by an adequate legal framework to protect public health by ensuring that perpetrators are sanctioned proportionally to the harm caused. National and international legal and regulatory regimes should ideally function as a coherent whole.

## A LEGAL LEVERS MATRIX

We propose a “legal levers matrix” to help translate WHO’s SF definition into national and international legal frameworks. Although the WHO definition is not *per se* legally binding, member states can draw from it to adapt their own laws and to harmonize provisions across countries.

To protect individuals and communities from SF medicines, countries should adopt both *administrative actions*—which do not need to be punitive and can focus on organizational and professional regulation to correct weaknesses in the quality systems—and civil and criminal actions—which may result in liabilities that are retributive and/or compensatory. Civil action may apply in the absence of proven intent, resulting in *civil liability for substandard medicines*, which is essentially compensatory in character. Criminal action may apply when there is evidence of fraudulent intent or gross negligence (including intentional adoption of risky procedures), resulting in *criminal liability for falsified products*. Legal levers should apply to natural and legal persons and be commensurate to factors such as severity (e.g., fatal, life-threatening, and long-term sequelae), size (e.g., number of victims) of the harm caused, and nature/size of the potential harm (in case the medicine is detected and confiscated before reaching and harming people).

National systems should be supported by a set of international policies and guidelines oriented toward attaining the sustainable development goals, including UHC. A number of transnational legal and policy frameworks exert an influence over the shaping and implementation of national systems, such as human rights laws, health regulations, and trade agreements among others (Figure 1).

## CONCLUSION

Universal health coverage cannot be achieved without equitable access to quality medicines; the SF medicine market is fed by failure to ensure access to health. Appropriate legal frameworks are integral part of the actions needed to safeguard patients’ rights to health.

Substandard and falsified medicines are at the crossroad between public health and regulations/law: they are a global public health problem, calling for a global response and for directions in the governance of regulatory and enforcement activities to take place in countries. This would require, for instance, that member states endeavor to align their civil and criminal legislations and to coordinate their implementation and continue to pursue regulatory harmonization,^19,20^ enhance collaboration across legal and regulatory authorities between LMICs and high-income countries, and apply strict regulatory oversight for medicines manufactured for export only.

We recognize that developing and operationalizing our proposed legal levers matrix would be a complex undertaking, between inherent difficulties (e.g., proving the intent and proving the potential harm) and institutional barriers, conflicting interests, and differing stakeholders’ priorities. Nonetheless, the growing awareness that SF medicines may hamper the attainment of UHC creates a momentum for developing and promoting new instruments to fight SF medicines. We bring this to the attention of an audience of medical and public health professionals to foster dialogue and coordination among public health and law stakeholders.

Our public health-informed “legal levers matrix,” based on a consistent interpretation of the WHO definition and on the intentionality and level of harm caused, needs to be nested within a supportive culture of global governance. This means that international organizations (and funders) must support member states in developing and implementing appropriate configurations of the matrix to reduce the problem of SF medicines and to realize the goal of UHC.

We do not presume to answer all questions around medicines’ quality. For instance, related important issues that could not be addressed here are the pertinence of standards and specifications in pharmacopoeias and good practices guidelines, which are less straightforward than generally thought, as products may fail or succeed depending on the criteria applied, the transparency on efficacy/safety data from clinical development, and issues related to corruptive practices in the pharmaceutical sector.

The issues at stake must be seen against the interplay between societies, economies, and behaviors. Until and unless medicines cease being dealt with as a simple “commodity” instead of a “public good” and become available and affordable as quality-assured formulations to all layers of society, in poorly regulated contexts, there will be always incentives for negligently or criminally offering poor-quality, dangerous products.
